# Sex is not an independent predictor of 90-day outcomes after mechanical thrombectomy in Chinese patients with acute ischemic stroke: a prospective cohort study

**DOI:** 10.3389/fsurg.2025.1708117

**Published:** 2026-01-12

**Authors:** Quan Liu, Sijia Liu, Jialin Yang, Qing He

**Affiliations:** 1 Emergency Department, West China School of Medicine, Sichuan University, Sichuan University Affiliated Chengdu Second People's Hospital, Chengdu Second People's Hospital, Chengdu, China; 2 College of Pharmacy, Chengdu University of Traditional Chinese Medicine, Chengdu, Sichuan, China; 3 Clinical Laboratory, School of Clinical Medicine, Chengdu Medical College; The First Affiliated Hospital of Chengdu Medical College, Chengdu, China

**Keywords:** acute ischemic stroke, functional outcomes, mechanical thrombectomy, predictor, sex

## Abstract

**Objective:**

To explore the impact of sex difference in the 90-day favourable outcome after mechanical thrombectomy(MT) with acute ischaemic stroke(AIS).

**Methods:**

We consecutively enrolled AIS patients treated with MT from the First Affiliated Hospital of Chengdu Medical College and Nanjing First Hospital between June 2015 to June 2022. According to the inclusion and exclusion criteria. Patients were divided into two groups by sex, and detailed demographic, laboratory examination, imaging examination and clinical data were collected. The primary outcome (favourable 90-day outcomes, mRS 0-2) was prospectively followed up. Univariate and multivariate logistic regressions were performed with SPSS 26.0.

**Results:**

Among 334 patients analyzed (198 male, 136 female), females were significantly older (median age: 76.0 vs. 68.5 years, *P* < 0.001), had higher admission glucose levels (7.3 vs. 6.5 mmol/L, *P* = 0.031), and higher prevalence of atrial fibrillation (47.1% vs. 26.3%, *P* = 0.001). Male patients had higher smoking rates (59.6% vs. 5.1%, *P* < 0.001). In univariate analysis, female sex was associated with lower odds of favorable outcome (OR 0.520, 95% CI 0.323–0.836, *P* = 0.007). However, after adjustment for age, baseline NIHSS, 24-hour NIHSS, mTICI 2b–3, hemorrhagic transformation, smoking, and fasting glucose, sex was not an independent predictor of 90-day favorable outcome (aOR 0.97, 95% CI 0.451–2.081, *P* = 0.939).

**Conclusion:**

Sex and 90-day favorable outcomes in Chinese patients with AIS undergoing MT are not independently associated.

## 1 Introduction

Mechanical thrombectomy (MT) is highly effective for acute ischemic stroke (AIS) caused by anterior large vessel occlusion (LVO), doubling the probability of favorable functional outcomes (1–4). It is now the standard of care for eligible patients (5). Despite advances in reperfusion therapies, disparities in stroke outcomes by sex have been increasingly recognized. Several studies report that women experience worse clinical outcomes after MT, including higher mortality and lower rates of functional independence (6–14). The MR CLEAN trial further indicated higher rates of serious adverse events among women (15). However, emerging evidence challenges this notion. A meta-analysis of seven randomized trials within the HERMES collaboration (*n* = 1,762; 47% women) found no significant effect of sex on functional outcome or treatment efficacy (16). Moreover, some studies suggest that women may achieve equal or even better recanalization rates (17). Critically, these findings derive predominantly from clinical trials and non-Chinese populations. Whether sex differences in favorable post-MT outcomes exist in real-world Chinese cohorts remains unestablished. Therefore, we conducted this study to investigate potential sex differences in favorable outcomes among Chinese AIS patients treated with MT.

## 2 Method

### 2.1 Study design and participants

This was a prospective, observational cohort study conducted at two tertiary stroke centers in China: the Department of Neurology, First Affiliated Hospital of Chengdu Medical College (Western China), and Department of Neurology, Nanjing First Hospital (Eastern China), from June 2015 to June 2022.

Patients were consecutively enrolled if they met the following inclusion criteria (PICO framework): (1) Age ≥18 years; (2) Onset of symptoms < 6 h; (3) Symptoms consistent with WHO diagnostic criteria for stroke, and exclusion of intracranial hemorrhage by brain CT/MRI; (4) Large vascular occlusion by Digital Subtraction Angiography (DSA); (5) Thrombectomy treatment in accordance with local guidelines. The exclusion criteria were as follows: (1) History of intracranial hemorrhage within 3 months. (2) Acute bleeding diathesis, including but not limited to, a platelet count <10  ×  10 12/L, or INR >1.7; (3) Known allergies to contrast agents, narcotic drugs, et al; (4) Blood glucose <2.8 or > 22.0 mmol/L.

The study protocol was approved by the institutional ethics committees of both hospitals. Written informed consent was obtained from all participants or their legally authorized representatives.

### 2.2 Data collection

All participants will undergo baseline assessments by Trained neurologists and follow-up evaluations at 90 days post-enrollment. Patient data will be collected using standardized forms. At baseline, we will document demographic information (e.g., gender, age), clinical characteristics (e.g., stroke subtype), medical history (e.g., hypertension), biochemical test results (e.g., blood glucose), and NIHSS scores (assessed at baseline and 24 h post-MT). At 90-day follow-up, all patients will complete face-to-face or telephone-based structured interviews to evaluate functional outcomes (modified Rankin Scale score, mRS) and survival status.

### 2.3 Clinical definitions and outcome

The primary outcome was favorable functional outcome at 90 days, defined as mRS score 0–2. Secondary outcomes included: (1) Successful reperfusion [post-procedure modified Thrombolysis in Cerebral Ischemia Scal(mTICI) grade ≥2b] (18); (2) 90-day all-cause mortality; (3) Hemorrhagic transformation (HT), defined as any intracranial hemorrhage on follow-up imaging (19).

Stroke etiology was classified according to the Trial of ORG 10172 in Acute Stroke Treatment (TOAST) criteria as: (1) large-artery atherosclerosis, (2) cardioembolism, or (3) other/undetermined etiology (20).

### 2.4 Statistical analysis

This study aimed to investigate the association between sex and outcomes following MT in patients with AIS. Patients were stratified by sex (male vs. female), and their baseline clinical characteristics were compared. Functional outcomes were assessed using the mRS at 90 days post-procedure. Based on these scores, patients were categorized into groups with favorable (mRS score 0–2) and unfavorable outcomes (mRS score 3–6). Univariate logistic regression was used to examine crude associations between sex and outcomes. Variables with *P* < 0.05 in univariate analysis or known prognostic importance were included in a multivariable logistic regression model to identify independent predictors of favorable outcome. The final model included: sex, age, smoking status, fasting glucose, baseline NIHSS, mTICI 2b–3, 24-hour NIHSS, and HT. Adjusted odds ratios (aORs) with 95% confidence intervals (CIs) were reported. A two-sided *P*-value < 0.05 was considered statistically significant.

In this study, continuous variables were presented as the mean (standard deviation, SD) or the median (interquartile range, IQR), depending on whether the data conformed to a normal distribution. Categorical variables were presented as percentages. The results were considered statistically significant at *P* < 0.05. All statistical analyses were performed using the SPSS software, version 26.0 (IBM Inc, Armonk, NY, USA).

## 3 Results

### 3.1 Baseline characteristics

**Figure 1:**
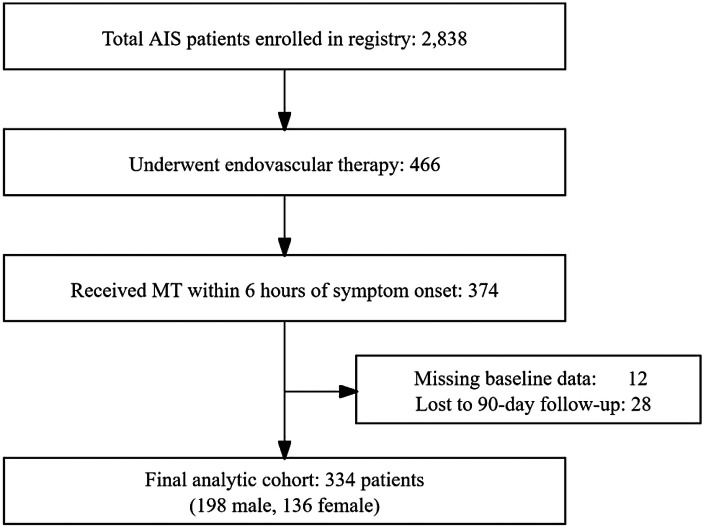
Participant flowchart.

Of 2,838 AIS patients enrolled in the registry, 466 underwent endovascular therapy; 374 received MT within 6 h of onset. After excluding 12 patients with missing baseline data (including incomplete NIHSS, glucose, or occlusion site information) and 28 patients lost to 90-day follow-up, a total of 334 patients were included in the final analysis (198 male, 136 female) (Figure 1). Compared to males, females were significantly older [median age: 76.0(67.5–83.0) vs. 68.5(60.0–79.0) years, *P* < 0.001], had higher fasting glucose [7.3(5.7–8.7) vs. 6.5 (5.3–8.0) mmol/L, *P* = 0.031], and higher prevalence of atrial fibrillation [64(47.1%) vs. 52(26.3%), *P* = 0.001], prior antiplatelet use [32(23.5%) vs. 29(14.6%), *P* = 0.039], and prior statin use [27(19.9%) vs. 23 (11.6%), *P* = 0.038]. Males had higher smoking rates [118(59.6%) vs. 7(5.1%), *P* < 0.001]. There were also differences in TOAST classification and occlusion site distribution (all *P* < 0.05) (Table 1).

**Table 1:** Baseline characteristics of included patients.

Characteristic	Male (*n* = 198)	Female (*n* = 136)	*P*-value
Age (years) (IQR)	68.5 (60.0–79.0)	76.0 (67.5–83.0)	0.000
Hypertension (%)	124 (62.6)	84 (61.8)	0.873
Diabetes (%)	39 (19.7)	29 (21.3)	0.717
Smoking (%)	118 (59.6)	7 (5.1)	0.000
Coronary artery disease (%)	33 (16.7)	27 (19.9)	0.456
Hyperlipidemia (%)	3 (1.5)	4 (4.4)	0.104
Atrial fibrillation (%)	52 (26.3)	64 (47.1)	0.001
Previous stroke (%)	38 (19.2)	34 (25.0)	0.205
Pre-antiplatelet (%)	29 (14.6)	32 (23.5)	0.039
Pre-statin (%)	23 (11.6)	27 (19.9)	0.038
TOAST classification (%)			0.000
LAA	88 (44.4)	30 (22.1)	
CE	84 (42.4)	85 (62.5)	
Other all	26 (13.1)	21 (15.4)	
Fasting glucose (IQR)	6.5 (5.3–8.0)	7.3 (5.7–8.7)	0.031
NIHSS score on admission (IQR)	15.0 (11.0–19.0)	16.0 (12.0–19.0)	0.295
Treatment with IV tPA (%)	77 (38.9)	45 (33.1)	0.279
24-hour NIHSS (IQR)	13.0 (5.0–20.0)	15.0 (9.0–20.0)	0.123
OTT min (IQR)	300.0 (210.0–385.0)	287.0 (210.0–351.0)	0.679
Occlusion site (%)			0.012
Anterior circulation	159 (80.3)	123 (90.4)	
Posterior circulation	39 (19.7)	139 (9.6)	

TOAST, trial of Org 10,172 in acute stroke treatment; LAA, large-artery atherosclerosis; CE, cardio embolism; IQR, interquartile range; NIHSS, National Institute of Health stroke Scale; OTT, onset treatmet time.

No significant differences were observed in hypertension, diabetes, coronary artery disease, or baseline NIHSS score.

### 3.2 Demographic and baseline characteristics of patients with different clinical outcomes

**Figure 2:**
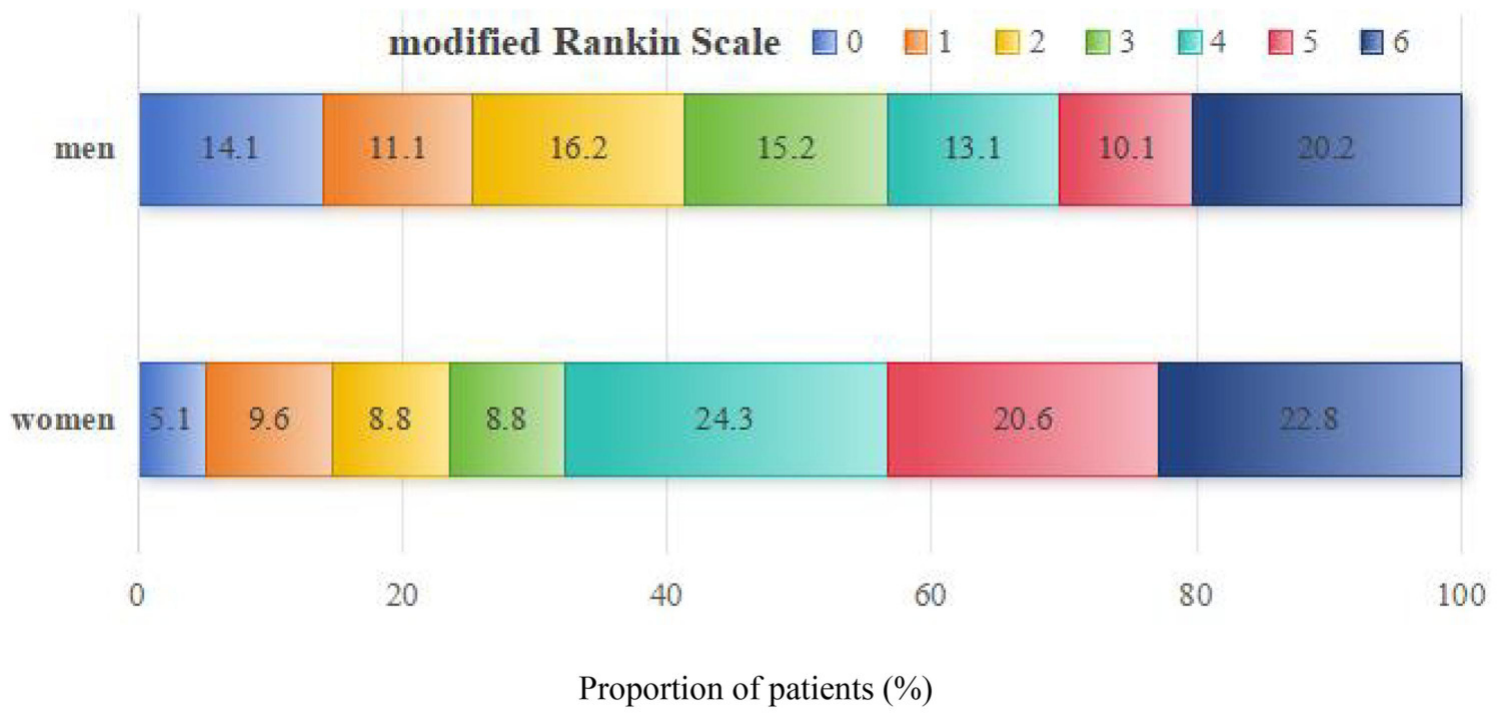
90-day mRS score distribution in both groups.

The cohort were included 117 patients in the favorable outcomes group (mRS 0–2) and 217 patients in the unfavorable outcomes group. The distribution of mRS scores at 90 days, stratified by sex, is presented in Figure 2. Compared with the unfavorable outcomes group (mRS 3-6), they exhibited a higher prevalence of male sex and smoking history, as well as superior recanalization rates (mTICI 2b-3) (*P* < 0.05). Additionally, the favorable outcomes group was characterized by a younger age, lower admission fasting glucose levels, and attenuated baseline NIHSS scores (*P* < 0.05). Furthermore, these patients demonstrated improved 24-hour NIHSS scores and a decreased rate of hemorrhagic transformation (*P* < 0.05) (Table 2).

**Table 2:** Demographic and baseline characteristics of patients with different clinical outcomes.

Characteristic	mRS 0-2 (*n* = 117)	MRS 3-6 (*n* = 217)	*P*-value
Age (years)	68 (60–77)	75 (66–82)	<0.0001
Male gender (%)	81 (69.23%)	117 (53.92)	0.007
Hypertension (%)	68/117 (58.12)	140/217 (64.52)	0.250
Diabetes (%)	21/117 (17.95)	47/217 (21.66)	0.422
Smoking (%)	60/117 (51.28)	65/217 (29.95)	<0.0001
Coronary artery disease (%)	22/117 (18.80)	38/217 (17.51)	0.769
Hyperlipidemia (%)	4/117 (3.42)	5/217 (2.30)	0.548
Atrial fibrillation (%)	40/117 (34.19)	76/217 (35.02)	0.879
Previous stroke (%)	19/117 (16.24)	53/217 (24.42)	0.083
Pre-antiplatelet (%)	19/117 (16.24)	42/217 (19.35)	0.482
Pre-statin (%)	16/117 (13.68)	34/217 (15.67)	0.626
Total cholesterol (IQR)	4 (3.4–5.0)	4.08 (3.5–5.0)	0.830
Fasting glucose (IQR)	6 (5.0–7.6)	7.09 (5.8–9.0)	<0.0001
TOAST classification (%)			0.116
LAA	49/117 (41.9)	69/217 (31.8)	
CE	56/117 (47.9)	113/217 (52.1)	
Other all	12/117 (10.3)	35/217 (16.1)	
NIHSS score on admission (IQR)	13 (12.0–18.0)	16 (13.0–21.0)	<0.0001
Treatment with IV tPA (%)	45/117 (38.5)	77/217 (35.5)	0.590
24-hour NIHSS (IQR)	5 (3–11.5)	16 (12–22.5)	<0.0001
OTT min (IQR)	300 (221–357)	287 (200–360)	0.260
Occlusion site (%)			0.701
Anterior circulation	100/117 (85.5)	182/217 (83.9)	
Posterior circulation	17/117 (14.5)	35/217 (16.1)	
mTICI 2b-3 (%)	109/117 (93.16)	179/217 (82.49)	0.007
Hemorrhagic transformation (%)	11/117 (9.4)	54/217 (24.9)	0.001

mTICI, modified Thrombolysis in Cerebral Ischemia Scal.

### 3.3 Univariate analysis of clinical outcomes and postoperative complications by Sex

In univariate analysis, female sex was associated with significantly lower odds of favorable outcome (OR 0.520, 95% CI 0.323–0.836, *P* = 0.007) (Table 3). There were no significant differences in successful reperfusion (OR 0.791, 95% CI 0.423–1.480, *P* = 0.464), 90-day mortality (OR 1.266, 95% CI 0.750–2.136, *P* = 0.378), or hemorrhagic transformation (OR 1.043, 95% CI 0.602–1.808, *P* = 0.881).

**Table 3:** Univariate analysis of sex and clinical outcomes in AIS patients with MT.

Clinical outcomes	Male (*n* = 198)	Female (*n* = 136)	OR (95%CI)	*P*-value
The primary outcome				
mRS score 0–2 at 90 days (%)	81 (40.9)	36 (26.5)	0.520 (0.323–0.836)	0.007
The secondary outcomes				
Successful reperfusion (%)	173 (87.4)	115 (84.6)	0.791 (0.423–1.480)	0.464
90-day mortality (%)	40 (20.2)	33 (24.3)	1.266 (0.750–2.136)	0.378
Hemorrhagic transformation (%)	38 (19.2)	27 (19.9)	1.043 (0.602–1.808)	0.881

mRS, modified rankin scale.

### 3.4 Independent predictors of 90-day functional outcomes post-MT

After adjustment for age, smoking, fasting glucose, baseline NIHSS, mTICI 2b–3, 24-hour NIHSS, and hemorrhagic transformation, female sex was not an independent predictor of 90-day favorable outcome (aOR 0.97, 95% CI 0.451–2.081, *P* = 0.939) (Table 4). The strongest independent predictor was 24-hour NIHSS (aOR 0.792 per point decrease, 95% CI 0.744–0.844, *P* < 0.001). The final model demonstrated good calibration (Hosmer-Lemeshow *χ*^2^ = 6.32, df = 8, *P* = 0.612) and moderate discrimination (AUC = 0.78).

**Table 4:** Mnivariate analysis of sex and clinical outcomes in AIS patients with MT.

Variables	aOR (95%CI)	*P*-value
Male	0.969 (0.451–2.081)	0.939
Age	0.984 (0.958–1.012)	0.257
Smoking	0.470 (0.216–1.021)	0.057
Fasting glucose	0.969 (0.870–1.080)	0.571
NIHSS score on admission	1.062 (0.996–1.133)	0.065
24-hour NIHSS	0.792 (0.744–0.844)	0.0001
mTICI 2b-3	0.524 (0.196–1.401)	0.198
Hemorrhagic transformation	2.015 (0.846–4.799)	0.114

## 4 Discussion

Our study demonstrates that although female sex is initially associated with worse 90-day functional outcomes after mechanical thrombectomy for AIS, this association disappears after adjusting for age, stroke severity, and procedural factors. Thus, sex itself does not independently predict outcome in this Chinese cohort.

Long term differences in hormonal profiles and lifestyle factors between males and females contribute to established sex disparities in stroke epidemiology, pathophysiology, treatment, and outcomes (21). Nevertheless, whether these differences influence prognosis after MT for AIS remains debated, particularly due to a scarcity of prospective cohort studies in Chinese populations and inconsistent findings across existing literature. For instance, studies on anterior circulation large vessel occlusion have reported higher rates of symptomatic intracranial hemorrhage and longer treatment delays in men, yet ultimately found no sex difference in final functional outcome (22). In contrast, investigations of posterior circulation (vertebrobasilar) occlusions have yielded conflicting results, with one study indicating worse short-term outcomes in women (23) and another reporting no sex-based differences (24).

Our analysis revealed significant disparities in demographic and clinical characteristics. Female patients were older (median age 76.0 years vs. 68.5 years in males) and exhibited a higher prevalence of atrial fibrillation and cardioembolic stroke etiology. Male patients, however, had higher rates of smoking history and large-artery atherosclerotic stroke. These findings align with previous reports (25, 26) and are consistent with the baseline profiles of female patients described in posterior circulation occlusion studies, which also noted advanced age and higher atrial fibrillation prevalence in women (27). The older average age of female stroke patients undergoing MT in our cohort may be linked to the sharp rise in stroke incidence among postmenopausal women and their longer average life expectancy. Pérez-Sánchez et al. (28) reported that women experienced longer onset-to-puncture times and were less likely to receive MT for AIS compared to men. In our study, we observed no statistically significant difference in onset-to-puncture time between sexes, a finding that contrasts with reports of longer treatment delays in men from other studies and may have contributed to the comparable functional outcomes achieved by women in our cohort.

A comprehensive meta-analysis of seven randomized trials from the HERMES collaboration found that patient sex did not significantly affect clinical outcomes or modify the treatment effect of thrombectomy when performed within 6 h of stroke onset (16). Corroborating this, Tan et al. (29) and the ANGEL-ACT registry study (24) both reported comparable functional outcomes between men and women with basilar artery occlusion undergoing MT. Further supporting evidence comes from a large-scale prospective multicenter cohort study (*n* = 2,316) of AIS patients treated with MT, which suggested that observed sex disparities in 90-day outcomes were attributable to confounding variables, particularly age and pre-stroke functional status, rather than biological sex (30). Notably, that study found no significant association between sex and the probability of achieving a favorable functional outcome at 90 days. Our results validate and extend these conclusions to a real-world Chinese population, thereby strengthening the generalizability of equivalent thrombectomy benefits across sexes and diverse ethnic groups, provided adequate adjustment for confounding factors is performed.

Multiple studies have consistently reported that female patients tend to experience poorer outcomes after MT for AIS, evidenced by higher mortality rates and less favorable functional recovery (10–13). Such disparities have been reported in studies of both anterior and posterior circulation strokes, though the findings are not unanimous. Our results contradict these observations, a discrepancy potentially explained by several factors. These include the tendency for female patients to be older, present with more severe strokes, and potentially receive less optimal treatment. Furthermore, interactions between occlusion site (anterior vs. posterior circulation), race, and sex may also influence post-thrombectomy outcomes (31, 32). Future research should prioritize elucidating sex-specific mechanisms, encompassing both biological and social determinants, and should incorporate stratified analyses by occlusion site to refine personalized treatment strategies further (33).

This study has several strengths and limitations. It represents one of the first prospective, multicenter cohort studies to evaluate sex differences in MT outcomes specifically in a Chinese population. The real-world design, standardized data collection, and substantial sample size enhance external validity and provide valuable evidence for sex-specific stroke care research. Limitations must also be acknowledged. First, incomplete documentation of critical time metrics, such as door-to-imaging and door-to-groin puncture times, may introduce residual confounding, despite all procedures being completed within the 6-hour therapeutic window; this limits deeper exploration of the sex-based differences in treatment delays observed in other studies. Second, pre-stroke functional status was not uniformly captured at enrollment, despite attempts to retrieve it during follow-up interviews. The lack of standardized pre-stroke mRS assessment may impair the precision of outcome interpretation, particularly for older patients with potential comorbidities or cognitive decline. In addition, loss to follow-up (*n* = 28, ∼7.5% of MT-treated patients) could introduce potential attrition bias. Third, the analysis did not include several potentially influential clinical and imaging variables, such as the specific occlusion site and baseline Alberta Stroke Program Early CT Score details. The inclusion of these variables is crucial for understanding the discrepant conclusions across studies, particularly those comparing anterior and posterior circulation strokes. Fourth, we did not collect data on hormonal status, menopausal stage, or hormone replacement therapy use, which are potentially significant biological variables in sex-specific outcome analyses. Future prospective studies should incorporate these parameters to better understand the biological mechanisms underlying sex differences in stroke outcomes. Fifth, the current sample size precluded meaningful subgroup analyses based on device type or procedural characteristics. Finally, as a *post hoc* analysis of prospective cohort data, the findings may be subject to inherent methodological biases. These limitations highlight important directions for future research aimed at optimizing sex-specific stroke care protocols.

## 5 Conclusions

In this prospective cohort of Chinese patients with AIS undergoing MT, female sex is not an independent predictor of 90-day functional outcome. Apparent disparities are attributable to age, stroke severity, and comorbidities. These findings support equitable delivery of thrombectomy services irrespective of sex. The findings of this study require further confirmation through large-scale, high-quality clinical research.

## Data Availability

The raw data supporting the conclusions of this article will be made available by the authors, without undue reservation.
